# Effect of the Combined Supplementation of Nucleotides and Mannan Oligosaccharides as Feed Additives in the Diet of *Penaeus vannamei* in a Synbiotic System

**DOI:** 10.3390/ani16121888

**Published:** 2026-06-18

**Authors:** Gênison Carneiro Silva, Scarlatt Paloma Alves da Silva, Danielle Alves da Silva, Hugo Rodrigo Monteiro de Queiroz Maia, Suzianny Maria Bezerra Cabral da Silva, Giovani Sampaio Gonçalves, João Fernando Albers Koch, Luis Otavio Brito

**Affiliations:** 1 Laboratório de Carcinicultura, Universidade Federal Rural de Pernambuco, Rua Dom Manoel de Medeiros, s/n, Dois Irmãos, Recife 52171-900, Pernambuco, Brazil; doutorado.danielle2020@gmail.com (D.A.d.S.); rodrigoqueirozmaia@gmail.com (H.R.M.d.Q.M.); luis.obsilva@ufrpe.br (L.O.B.); 2 Laboratório de Sanidade de Animais Aquáticos, Universidade Federal Rural de Pernambuco, Rua Dom Manoel de Medeiros, s/n, Dois Irmãos, Recife 52171-900, Pernambuco, Brazil; paloma.alves9521@gmail.com (S.P.A.d.S.); suzianny.silva@ufrpe.br (S.M.B.C.d.S.); 3 Laboratório de Nutrição em Aquicultura do Instituto de Pesca, Av. Abelardo Menezes, S/N acesso Rod, Washington Luís km 444, São José do Rio Preto 15025-620, São Paulo, Brazil; giovani.goncalves@sp.gov.br; 4 Biorigin, Rua XV de novembro, 865, Lençóis Paulista 18680-900, São Paulo, Brazil; joao.koch@biorigin.net

**Keywords:** *Bacillus*, economic efficiency, growth performance, gut health, *Vibrio*

## Abstract

The use of functional feeds in aquaculture has increased due to the reduction in fishmeal and the need to improve the gut health of farmed organisms. This study evaluated the effects of supplementing nucleotides (NT) and mannan oligosaccharide (MOS) in the diet of juvenile *Penaeus vannamei* reared in a synbiotic system for 60 days. Four treatments were tested: a control diet without additives (RC); a diet supplemented with 2 g NT kg^−1^ (NT); a diet supplemented with 2 g MOS kg^−1^ (MOS); and a diet supplemented with 1 g NT kg^−1^ and 1 g MOS kg^−1^ (NT/MOS). Water quality, zootechnical performance, gut morphology, microbiota indicators, and economic benefits were evaluated. There were no significant differences in water quality or production performance among treatments. However, additive supplementation improved economic efficiency and net revenue, with the NT/MOS treatment showing a 7.06% economic gain relative to the control. MOS supplementation increased the proportion of *Bacillus* spp. to 13.9 × 10^7^ CFU g^−1^, suggesting a prebiotic effect and potential control of *Vibrio* spp. Morphologically, supplemented shrimp exhibited shorter mucosal folds and thicker gut walls. NT and MOS supplementation appeared to be an economically promising strategy for shrimp production in synbiotic systems.

## Simple Summary

Shrimp farming increasingly relies on sustainable feeding strategies to maintain shrimp health and production efficiency while reducing dependence on traditional feed ingredients. Additives such as nucleotides (NT) and mannan oligosaccharide (MOS) have been investigated because of their potential to support gut health and microbial balance in cultured shrimp. In this study, juvenile *Penaeus vannamei* were reared in a synbiotic system and fed diets supplemented with nucleotides, MOS, or a combination of both additives for 60 days. Although growth performance and water quality were not significantly affected by the dietary treatments, shrimp receiving supplemented diets showed improved economic returns compared to those fed the control diet. In addition, MOS supplementation was associated with a higher abundance of beneficial *Bacillus* spp., suggesting a positive modulation of the gut microbiota and a possible reduction in opportunistic bacteria such as *Vibrio* spp. Histological observations also indicated changes in gut structure in shrimp fed additive-containing diets. These findings suggest that combining NT and MOS may represent a practical nutritional strategy to improve the sustainability and profitability of shrimp farming under synbiotic culture conditions.

## 1. Introduction

Shrimp farming, particularly *Penaeus vannamei*, has become one of the most important sectors of global aquaculture due to its high economic importance, with global production reaching 6.8 million tons in 2022 [1]. Commonly known as the Pacific white shrimp, this species is characterized by its rapid growth, tolerance to environmental fluctuations, strong osmoregulatory capacity, and adaptability to different culture systems, including autotrophic and heterotrophic production systems, such as biofloc and synbiotic systems [2,3,4].

Among recent technological advances in shrimp farming, synbiotic systems have gained considerable attention because they combine minimal water exchange with high production efficiency. In these systems, the aquatic microbiome plays a crucial role in nutrient recycling, pathogen suppression, and the maintenance of animal health [5,6,7]. Despite these advantages, maintaining microbial balance, preventing disease outbreaks, and optimizing shrimp performance remain major challenges [8,9,10].

Functional feed additives, such as nucleotides (NT) and mannan oligosaccharides (MOS), represent a promising strategy for microbial management in biofloc and synbiotic aquaculture systems. NT are essential biomolecules involved in nucleic acid synthesis, cellular metabolism, and energy transfer. These compounds have been reported to enhance gut development, immune function, and stress resistance [11,12,13]. In the gut tract, nucleotides promote epithelial regeneration and maintain mucosal integrity, thereby enhancing nutrient absorption [12,14,15]. Furthermore, studies have shown that nucleotide supplementation can stimulate the innate immune response in shrimp by increasing hemocyte production and enhancing the activity of enzymes such as phenoloxidase, which plays a key role in pathogen defense [15,16]. Previous studies have demonstrated that dietary supplementation with nucleotides (0.15–0.3 g NT kg^−1^ feed) in *P. vannamei* reared at a density of 100 shrimp m^−2^ in a synbiotic system improved growth performance, modulated gut microbiota, and increased economic returns [17].

MOS, derived from yeast cell walls, function as prebiotics by modulating the gut microbiota and promoting shrimp health and immune competence [18,19,20]. These compounds promote the proliferation of beneficial bacterial populations, such as *Lactobacillus* and *Bifidobacterium* spp., which compete with pathogenic microorganisms. In addition, improved feed digestibility and nutrient utilization associated with MOS supplementation may contribute directly to enhanced shrimp performance [18,20,21,22,23]. Previous studies have demonstrated that dietary supplementation with solubilized MOS (1 g kg^−1^ feed) in *P. vannamei* reared at a density of 100 shrimp m^−2^ in a synbiotic system improved growth performance, modulated gut microbiota, and increased economic returns [24].

However, to date, no studies have evaluated the effects of combined dietary supplementation with NT and MOS in synbiotic culture systems for *P. vannamei*. The interaction between dietary supplementation and environmental microbial management may enhance shrimp growth, survival, and the abundance of beneficial microbial populations while improving the economic efficiency of shrimp production. Therefore, this study aimed to evaluate the effects of combined dietary supplementation with NT and MOS on water quality, production performance, gut microbiota, gut histomorphology, and economic performance of *P. vannamei* reared in a synbiotic aquaculture system.

## 2. Materials and Methods

### 2.1. Experimental Design and Culture Conditions

The experiment was conducted at the Laboratório de Carcinicultura (LACAR) of the Department of Fisheries and Aquaculture (DEPAq), Federal Rural University of Pernambuco. The experimental period lasted 60 days and included four treatments with four replicates each in a completely randomized design, totaling 16 experimental units distributed among the following treatments: a control diet without additives (RC); a diet supplemented with 2 g NT kg^−1^ (NT); a diet supplemented with 2 g MOS kg^−1^ (MOS); and a diet supplemented with 1 g NT kg^−1^ and 1 g MOS kg^−1^ (NT/MOS). The dietary inclusion levels were selected based on previous studies conducted at LACAR by da Silva et al. (2025) [17].

Experimental units consisted of fiberglass tanks with a useful volume of 800 L. Each tank received 200 L of biofloc inoculum obtained from the nursery system, with the following water quality characteristics: TAN = 0.4 mg L^−1^, NO_2_-N = 0.5 mg L^−1^, NO_3_-N = 20 mg L^−1^, pH = 7.4, settleable solids = 9.0 mL L^−1^, and alkalinity = 140 mg CaCO_3_ L^−1^. Subsequently, 600 L of seawater (24 g L^−1^ salinity), previously disinfected with 40 mg L^−1^ active chlorine supplied as sodium hypochlorite, was added to each tank. After chlorination, the water was dechlorinated by aeration for 48 h.

For preparation of the synbiotic mixture, the following ingredients were added per cubic meter of culture water: 5 g of rice bran, 0.5 g of sugar, 0.25 g of *Lithothamnium* (Lothar, Primasea, Candeias, BA, Brasil), 0.045 g of a commercial probiotic containing *Bacillus subtilis*, *B. licheniformis*, *B. amyloliquefaciens*, *B. megaterium*, and *B. pumilus* (at a concentration of 10^9^ CFU g^−1^) (Proquatic^®^ PondPlus^®^, MSD Animal Health, Rahway, NJ, USA), and 50 mL of dechlorinated seawater (24 g L^−1^ salinity).

The synbiotic mixture was prepared according to the protocol described by da Silva et al. (2025) [17], following these steps: (i) activation phase—the probiotic was combined with sugar and water and aerated for 2 h; (ii) anaerobic phase—rice bran and *Lithothamnium* were added, after which aeration was discontinued and the mixture remained under anaerobic conditions for 24 h; (iii) aerobic phase—following the anaerobic period, aeration was resumed and maintained for an additional 24 h. After completion of the three preparation stages, the synbiotic mixture was applied to the culture tanks. Throughout the study, the synbiotic was applied every three days and discontinued once settleable solids reached 10 mL L^−1^, system maintenance. System maintenance after settleable solids reached 10 mL L^−1^ was performed by applying only the first stage of the synbiotic preparation at 5-day intervals.

To maintain total alkalinity above 100 mg CaCO_3_ L^−1^ and pH near 7.0, 20 g m^−3^ of calcium and magnesium hydroxide mixture was applied every five days throughout the experimental period. No water exchange was carried out during the experimental period; only water lost through evaporation was replenished with dechlorinated freshwater.

### 2.2. Experimental Diet

The experimental diets were produced by the Advanced Center for Inland Fisheries, Institute of Fisheries, located in São José do Rio Preto, São Paulo, Brazil, following standard shrimp-feed manufacturing procedures. Diets were formulated to meet the nutritional requirements of *P. vannamei*. The control diet was prepared without additives, whereas the remaining experimental diets were supplemented with NT and MOS (Table 1). The NT source consisted of a commercial product containing 15% RNA (Biotide Extra^®^, Biorigin, São Paulo, SP, Brazil), whereas the MOS source consisted of a commercial additive containing 27.2% mannan oligosaccharides (Hypergen^®^, Biorigin, São Paulo, SP, Brazil).

### 2.3. Shrimp Source and Stocking

*P. vannamei* postlarvae (PL10; initial weight 0.003 g) were obtained from a commercial hatchery facility (Aquatec, RN, Brazil) and initially reared in fiberglass tanks with a usable capacity of 800 L. During this nursery phase, the PL were stocked at a density of 2500 PL m^−3^ and reared under nursery conditions until reaching an average body weight of 3.0 ± 0.04 g. Once the shrimp reached the target weight, they were carefully transferred to the experimental units, where they were stocked at 80 shrimp m^−3^ (equivalent to 100 shrimp per tank).

### 2.4. Water Quality

Water quality variables monitored daily included dissolved oxygen (DO) and temperature (AT-160, Microprocessor-Controlled Oximeter, AlfaKit, Florianópolis, SC, Brazil) at 8 a.m. and 4 p.m.; every five days, pH (AK90, Asko, RS, Brazil), salinity (AZ86031 salinometer, AZ Instrument Corp., Taichung, Taiwan), and settleable solids (SS; Imhoff cone) were measured [25]; every 10 days, total ammonia nitrogen (TAN), nitrite nitrogen (NO_2_-N), and total alkalinity were determined [25]; every 20 days, nitrate nitrogen (NO_3_-N) and orthophosphate were determined [26].

### 2.5. Feed Management, Shrimp Performance, and Economic Benefits

Throughout the experimental period, the shrimp were fed three times a day (at 8:00 a.m., 1:00 p.m., and 4:00 p.m.) with the diets described in Table 1. The feeding rate was initially set at 4.5% of biomass and gradually reduced over the 60-day culture period to 2.7%. Feeding rates were adjusted according to the estimated consumption and mortality of the culture units; to this end, partial biometric assessments (*n* = 30 samples) were taken every 10 days to assess the shrimp’s weight gain and survival [27]. On days 30 and 60 of the experimental period, a complete biometric assessment was conducted, in which all shrimp were weighed and counted to assess the following parameters: biomass gain (final biomass (g)—initial biomass (g)); final body weight (final biomass (g)/number of individuals at the end of the culture); feed conversion ratio (FCR) (amount of feed provided/biomass gain); survival (number of individuals at the end of cultivation/initial number of stocked individuals × 100) and yield (final biomass (kg)/volume of the experimental unit (m^3^)).

For the economic benefits, partial operating costs were considered, including expenses for feed and additives, as well as profitability indicators such as net profit, return on investment (ROI) [28], and relative difference (%) [29]. All calculations were standardized for a production area of 1 ha and a single production cycle. Production estimates were based on the shrimp performance obtained in the present experiment, including final weight, feed conversion ratio (FCR), and survival rate. Only feed and additive costs were included in the economic analysis. Fixed and variable operating costs (purchase of postlarvae, labor, electricity, fertilization, and alkalinity management, among others) were not included in the analysis, as they were the same for all treatments.

The following market prices were considered: commercial feed, USD 0.92 kg^−1^; MOS additive (Hypergen^®^, Biorigin, São Paulo, SP Brazil), USD 9.00 kg^−1^; NT additive (Biotide^®^, Biorigin, São Paulo, SP, Brazil), USD 7.60 kg^−1^; and shrimp selling price in Brazil, USD 3.70 kg^−1^. The exchange rate considered was 1 USD = BRL 5.22 (4 March 2026).

The economic performance of the treatments was compared using the following equations [28,29]:

Harvested shrimp (number) = Stocking density (shrimp ha^−1^) × Production area (ha) × Survival rate (%);

Harvested biomass (kg ha^−1^) = Harvested shrimp (number) × Final weight (kg);

Revenues (USD ha^−1^) = Harvested biomass (kg ha^−1^) × USD 3.70 kg^−1^;

Feed supplied = Harvested biomass (kg ha^−1^) × FCR;

Feed Cost (USD ha^−1^) = Feed Supplied (kg ha^−1^) × Feed Price (USD kg^−1^);

Additive Cost (USD ha^−1^) = (Feed Supplied (kg ha^−1^) × Inclusion (g/kg) × MOS Price (USD kg^−1^)/1000;

Additive Cost (USD ha^−1^) = (Feed Supplied (kg ha^−1^) × Inclusion (g/kg) × NT Price (USD kg^−1^)/1000;

Expenses (USD ha^−1^) = (Feed Cost (USD ha^−1^) + Additive Cost (USD ha^−1^));

Net income (USD ha^−1^) = Revenues (USD ha^−1^) − Expenses (USD ha^−1^);

Difference in net income (USD ha^−1^) = Net profit (USD ha^−1^) (MOS Treatment) − Net profit (USD ha^−1^) (Control);

ROI = [(Net profit from the additive treatment − Net profit from the control treatment)/Cost of the additive)] × 100

Relative difference (%) = [(Net profit from the additive treatment − Net profit from the control treatment)/Net profit from the control treatment] × 100

### 2.6. Presumptive Microbial Counts

Presumptive microbial counts of the shrimp gut were performed at the beginning, middle, and end of the experimental period. For each treatment, nine shrimp (three shrimp per tank) were sampled and pooled according to tank (approximately 50 mg per pool) [30]. Selective culture media (Mannitol Egg Yolk Polymyxin (MYP) agar, Sabouraud dextrose agar, and Thiosulfate Citrate Bile Sucrose (TCBS) agar) were used to enumerate target microbial groups, *Bacillus* spp., yeasts, and *Vibrio* spp. respectively, in the gut of the shrimp. Following anesthesia and euthanasia, shrimp were surface-disinfected by sequential immersion in 70% ethanol for 15 s, followed by a 1.5% sodium hypochlorite solution containing 0.1% Tween-80 for 15 min, and subsequently rinsed with distilled water. After disinfection, approximately 100 mg of gut tissue was aseptically collected. The samples were transferred to 9 mL of 0.1% buffered peptone water, homogenized, and serially diluted (1:10) in sterile saline solution (3%).

Aliquots of the dilutions were inoculated in triplicate onto Petri dishes containing TCBS agar, MYP agar, and Sabouraud dextrose agar for the enumeration of *Vibrio* spp., *Bacillus* spp., and yeasts, respectively. The inoculated plates were incubated at 30 °C for 24 h for bacterial quantification, while fungal cultures were incubated at 36 °C for 72 h [31]. Microbial abundance was expressed as colony-forming units (CFU) per gram of gut tissue, calculated according to the following equation:

CFU/g = (number of colonies × dilution factor)/weight of the gut sample.

### 2.7. Gut Histomorphology of Penaeus vannamei

**Figure 1:**
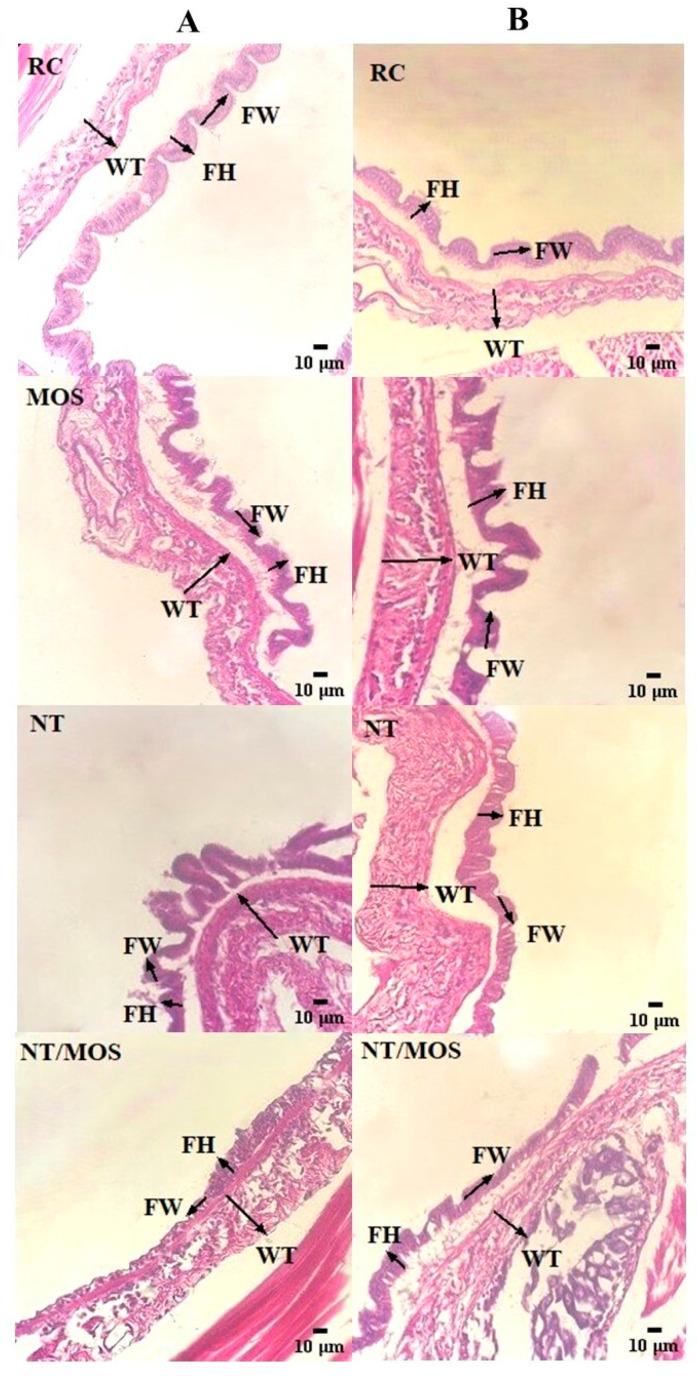
Histomorphology of the gut of *P. vannamei* fed the experimental diets. Column (**A**): anterior gut; (**B**): midgut. FH: mucosal fold height, FW: mucosal fold width, WT: gut wall thickness. Scale bar = 10 μm; Original magnification ×400.

The effects of the dietary supplementation on the gut mucosal morphology of *P. vannamei* were evaluated through histomorphology analyses performed on three shrimp collected from each experimental unit at the end of the trial, totaling 12 shrimp per treatment. After collection, the gut was aseptically excised and fixed in Davidson’s solution (AFA) for 72 h. Subsequently, the samples were transferred to 70% ethanol for histological processing and preparation of slides stained with hematoxylin and eosin (H&E) [32]. Histological sections were examined under a light microscope at 400× magnification. Histomorphometric evaluations were carried out using ImageJ software (v1.46r, National Institute of Health, Bethesda, MD, USA) on one section from the anterior and mid gut of each shrimp. For each shrimp, ten gut folds were measured to determine the following parameters: (1) mucosal fold height (FH); (2) mucosal fold width (FW); and (3) gut wall thickness (WT) (Figure 1). Histological slides were analyzed in a blinded manner, without the observer knowing the treatment allocation of each sample.

### 2.8. Statistical Analysis

Statistical analyses were performed using BioEstat 5.0 software (Instituto Mamirauá, Tefé, AM, Brazil). Data normality was assessed using the Shapiro–Wilk test (*p* < 0.05). Zootechnical performance variables were analyzed using one-way analysis of variance (ANOVA). Temporal variations in nitrite, nitrate nitrogen, and orthophosphate concentrations were analyzed using repeated-measures ANOVA, including treatment, time, and treatment × time interaction effects. For variables that did not meet normality assumptions (dissolved oxygen, temperature, pH, salinity, settleable solids, alkalinity, and TAN), the Friedman test was applied. Presumptive counts of *Vibrio*, *Bacillus*, and yeast, as well as gut histomorphological variables, were analyzed using the nonparametric Kruskal–Wallis test (*p* < 0.05), followed, when appropriate, by Student–Newman–Keuls or Dunn post hoc tests (*p* < 0.05). Results are presented as means with their respective 95% confidence intervals.

## 3. Results

### 3.1. Water Quality

Water quality variables measured during the experimental period are presented in Table 2. Dissolved oxygen concentrations remained above 5.0 mg L^−1^ throughout the study, whereas water temperature averaged approximately 27 °C and salinity remained stable at approximately 24 g L^−1^. pH remained stable at approximately 7.6, total alkalinity ranged from 150 to 159 mg CaCO_3_ L^−1^, settleable solids ranged from 5.6 to 6.4 mL L^−1^, and orthophosphate concentrations ranged from 16 to 18 mg L^−1^. No significant treatment effects were detected for any of these variables (*p* > 0.05). Regarding inorganic nitrogen compounds, TAN concentrations ranged from 0.15 to 0.39 mg L^−1^, NO_2_-N concentrations from 0.41 to 0.56 mg L^−1^, and NO_3_-N concentrations from 269 to 326 mg L^−1^, with no significant treatment effects observed among treatments (*p* > 0.05).

### 3.2. Shrimp Performance and Economic Benefits

**Figure 2:**
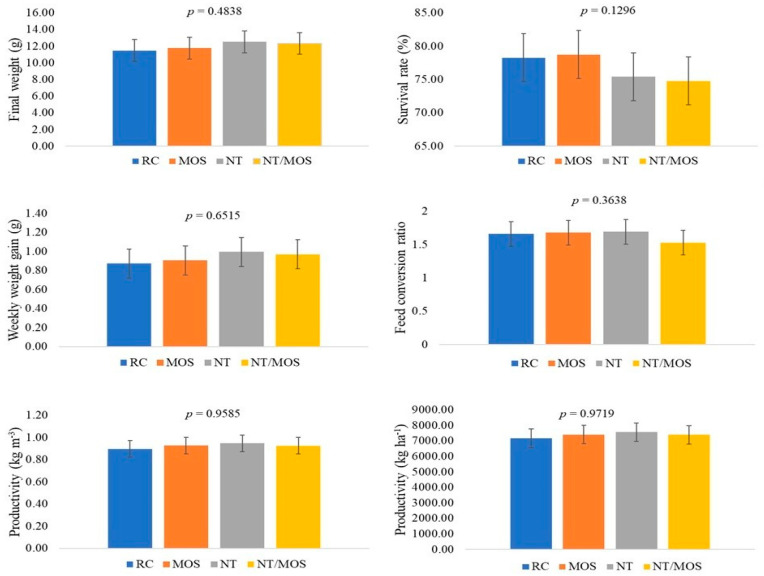
Shrimp performance fed experimental diets containing nucleotides and MOS during 60 days of culture in a synbiotic system. A control diet without additives (RC); a diet supplemented with 2 g NT kg^−1^ (NT); a diet supplemented with 2 g MOS kg^−1^ (MOS); and a diet supplemented with 1 g NT kg^−1^ and 1 g MOS kg^−1^ (NT/MOS).

Performance variables measured during the 60-day experimental period are presented in Figure 2. Survival exceeded 70%, whereas feed conversion ratio (FCR) values remained below 1.7 across all treatments, with no significant treatment effects detected (*p* > 0.05). Final weight, productivity, and weekly growth rate did not differ significantly among treatments (*p* > 0.05).

Return on investment (ROI) was estimated considering a hypothetical production area of 1 ha. The MOS, NT, and NT/MOS treatments yielded ROI values of 58.02, 207.49, and 588.97, respectively, relative to the control treatment. Additional net revenue relative to the control ranged from USD 130 to USD 1104 ha^−1^, resulting in a relative difference (%) between 0.83% and 7.06% (Table 3).

### 3.3. Presumptive Counts of Vibrio spp., Bacillus spp., and Yeast in the Shrimp Gut Tract

Presumptive counts of *Vibrio* spp., *Bacillus* spp., and yeasts determined on TCBS, MYP, and Sabouraud dextrose agar at days 0, 30, and 60 are presented in Table 4. Initial presumptive counts of *Vibrio* spp. revealed exclusively sucrose-fermenting colonies, with a total abundance of 5.14 × 10^7^ CFU g^−1^. At days 30 and 60, non-sucrose-fermenting colonies were also detected, although at substantially lower abundances. At the end of the experimental period, *Vibrio* spp. counts ranged from 0.05 × 10^7^ CFU g^−1^ in the NT/MOS treatment to 2.16 × 10^7^ CFU g^−1^ in the RC treatment. However, no significant differences were observed among treatments (*p* > 0.05).

*Bacillus* spp. had an initial count of 2.86 × 10^7^ CFU g^−1^ and increased throughout the culture period, with a final count ranging from 4.42 × 10^7^ (NT) to 13.9 × 10^7^ (MOS) CFU g^−1^, with significantly higher counts in the MOS treatment than in the remaining treatments (*p* = 0.0184). Yeasts had initial counts of 0.09 × 10^7^ CFU g^−1^, with counts increasing throughout the experimental period to 0.117 × 10^6^ (NT) and 0.164 × 10^6^ (MOS) CFU g^−1^; however, no significant treatment effects were detected (*p* > 0.05).

### 3.4. Shrimp Gut Histomorphology

Histomorphological measurements of the anterior and mid gut are presented in Table 5. Shrimp receiving NT- and MOS-supplemented diets exhibited anterior gut mucosal fold thickness values similar to or lower than those observed in the control treatment. Shrimp fed the MOS-supplemented diet exhibited greater mucosal fold height in the mid gut than those in the control treatment (Figure 1). Shrimp receiving dietary additives exhibited greater gut wall thickness values than those observed in the control group.

## 4. Discussion

Synbiotic systems are notable for their ability to create a microbiologically balanced environment, promoting the efficient recycling of nitrogen compounds within the aquaculture system [4,7,33]. The incorporation of organic substrates, such as vegetable meal, stimulates the proliferation of beneficial microbial communities, leading to improvements in water quality and system stability. This microbiota plays a fundamental role in reducing nitrogen compounds, minimizing their toxicity and maintaining suitable conditions for shrimp farming [34].

In the present study, water quality parameters remained within the recommended ranges for *P. vannamei* farming [35], reinforcing the efficiency of the synbiotic system. Furthermore, the inclusion of NT and MOS in the shrimp diets did not significantly influence water quality variables, as similar values were observed across treatments.

The results indicate no significant differences in the shrimp performance among the treatments, with survival rates exceeding 70% and feed conversion ratios below 1.7. Although growth performance did not differ significantly among treatments, additive-supplemented groups consistently exhibited numerically higher final weight and productivity values. While these differences were insufficient to demonstrate statistical significance, they translated into greater harvested biomass and revenue at the production scale. Because the economic analysis integrates multiple production variables simultaneously, including biomass yield, survival, feed conversion, and feed costs, these numerical improvements resulted in higher net revenue and ROI, particularly in the NT/MOS treatment. The absence of significant responses may be related to the inherent efficiency of synbiotic systems, which maintain stable microbial communities, promote nutrient recycling, and suppress opportunistic pathogens. Under these conditions, shrimp may already be reared close to their physiological optimum, thereby reducing the magnitude of responses to dietary supplementation [5,6], which may reduce the additional effects of additives. There are few studies on the synbiotic system that focus on the use of additives, as it is believed that the combination of probiotics and prebiotics (plant bran) already promotes a microbiota capable of maintaining good water quality and controlling pathogenic bacteria such as the genus *Vibrio* spp., since the constant introduction of probiotic bacteria throughout the culture period promotes competition among bacteria in the aquatic environment and in the animals’ gut [4,36]. Furthermore, the synbiotic system has shown good results in terms of the performance of *P. vannamei*, with growth rates, survival rates, and feed conversion ratios similar to those of systems such as biofloc and recirculation systems [5,17,37].

Another possible explanation for the absence of significant treatment effects relates to the supplementation levels evaluated in this study. The inclusion rates tested may not represent the optimal dietary concentrations under synbiotic culture conditions. Previous studies have reported beneficial effects of NT/MOS supplementation in aquatic species, including improved digestibility, enhanced disease resistance, increased feed intake, and greater weight gain [17,18,38,39,40]. For example, Rairat et al. (2022) [15] reported improvements in growth performance and immune responses of *P. vannamei* when dietary nucleotides were supplied at levels of 4–5 g kg^−1^ feed, whereas Abdel-Rahim et al. (2021) [16] observed positive effects of nucleotide supplementation under heat stress conditions. Similarly, Novriadi et al. (2024) [20] demonstrated improvements in growth performance and pathogen resistance with MOS inclusion levels ranging from 2 to 4 g kg^−1^ feed. Collectively, these findings suggest that the supplementation levels evaluated in the present study may have been insufficient to elicit measurable responses under the culture conditions tested.

Nucleotides participate in essential cellular processes, including nucleic acid synthesis, energy metabolism, and protein production, which may contribute to improved growth performance, health status, and physiological resilience under specific culture conditions [12]. Meanwhile, MOS may selectively stimulate beneficial microbial populations and contribute to gut health through the production of microbial metabolites and competitive exclusion of opportunistic bacteria [18,41,42]. However, most of these studies were conducted in conventional systems, typically in clear water, where the effects of additives tend to be more evident. Additionally, the combined supplementation strategy (1 + 1 g kg^−1^) may have elicited responses different from those obtained with the individual additives. This finding indicates that combining functional feed additives does not necessarily enhance their effects and that such interactions may depend on the culture environment, particularly in synbiotic systems.

In economic terms, the highest gross and net revenues were observed in the treatments that included NT and MOS, showing increases in net revenue of US$ 130 ha^−1^ (MOS), US$ 403 ha^−1^ (NT), and US$ 1104 ha^−1^ (NT/MOS) compared to the control (RC). Considering only variable costs (fixed costs excluded as they are constant across treatments), ROI values ranged from 58.02 to 588.97, with relative differences (%) ranging from 0.83 to 7.06%, making them more economically efficient. These data corroborate another study that evaluated different nucleotide dosages (75, 150, and 300 mg kg^−1^ of feed) and found higher net revenues and ROI with the use of these additives in *P. vannamei* production [17]. Economic analyses integrating biomass production, shrimp growth, feed conversion ratio, and feed additive costs are particularly relevant because economic constraints often limit the adoption of functional feed additives in commercial aquaculture.

The reduction in mucosal fold height, associated with increased gut wall thickness, particularly in the treatments supplemented with NT and MOS, may reflect gut remodeling in response to dietary supplementation. Gut morphology results varied across segments and treatments. In the foregut, MOS and NT/MOS reduced mucosal fold height, while NT showed intermediate values. In the midgut, however, only MOS increased the height of the mucosal folds. Gut wall thickness increased in all treatments, with higher values in the NT/MOS treatment in the foregut and in the MOS treatment in the midgut. Increased mucosal fold height has been associated with greater absorptive surface area and improved gut barrier function in aquatic organisms [43], whereas in the foregut, it acts primarily in food processing and transport [44]. However, the responses observed in the present study did not follow this pattern, possibly associated with the concentrations used and the interaction between the additives. Studies have shown that supplementation with MOS and NT produces a dose-dependent response, in which moderate levels promote improvements in gut integrity, while higher or lower levels may reduce mucosal height [21,45,46,47]. Studies conducted with *P. vannamei* showed that the inclusion of additives or bioactive dietary components can enhance local immune activation and the rate of cell turnover, resulting in thickening of the gut wall and structural changes that do not necessarily imply a reduction in digestive efficiency [48,49,50].

Thus, despite the reductions in mucosal fold height observed in some supplemented treatments, no adverse effects on growth performance, survival, or feed conversion ratio were detected under the conditions evaluated. These morphological responses occurred concurrently with increased gut wall thickness, suggesting gut remodeling rather than functional impairment. Because enterocytes, the primary cells responsible for nutrient absorption, are components of the gut mucosa and contribute to the overall structure of the gut wall [51], the observed increase in gut wall thickness indicates that structural modifications involved multiple tissue layers rather than changes in mucosal fold height alone. The stability of production parameters suggests that these morphological changes were not associated with measurable reductions in productive performance. Nevertheless, the biological significance of these gut modifications cannot be fully determined from morphometric measurements alone and should be further investigated through direct assessments of digestive physiology, nutrient absorption, and gut function.

To achieve optimal production performance and improve the nutritional efficiency of aquafeeds, maintaining a balanced gut microbiota is essential, as the intestine plays a central role in digestion and nutrient absorption [52]. Pathogenic bacteria, including *Vibrio* spp., may disrupt microbial homeostasis and impair gut health, potentially affecting the structure of the gut mucosa and the efficiency of nutrient utilization [53,54].

In the present study, the estimated microbial counts at the end of the 60-day culture period revealed significantly higher *Bacillus* spp. abundance in the MOS treatment compared with the other treatments. This finding supports the prebiotic activity of MOS and its ability to promote beneficial bacterial populations within the gut microbiota [4,17,18]. In addition, MOS can bind to lectin-like receptors present on the surface of *Vibrio* spp., potentially reducing bacterial adhesion to the gut epithelium. This mechanism was previously described by Butt et al. [18], who reported reduced shrimp mortality under conditions of elevated pathogen pressure. Lower *Vibrio* spp. counts were also observed in the NT/MOS treatment compared with the control treatment; however, these differences were not statistically significant. Therefore, although the results suggest that MOS supplementation may contribute to a more favorable gut microbial profile, the mechanisms through which NT and MOS interact to influence gut microbial communities and host physiology remain unclear and warrant further investigation.

## 5. Conclusions

The supplementation of nucleotides (NT) and mannan oligosaccharides (MOS) in the diets of *P. vannamei* reared in a synbiotic system did not affect water quality, shrimp growth performance, survival, or the feed conversion ratio. However, additive-supplemented treatments, particularly the NT/MOS combination, yielded higher estimated economic returns than the control treatment, suggesting improved production efficiency from an economic perspective. From a histological standpoint, dietary supplementation was associated with reduced gut mucosal fold height and increased gut wall thickness. These findings suggest structural remodeling of the gut without negative effects on productive performance. Furthermore, the MOS treatment promoted significantly higher *Bacillus* spp. counts than the other treatments, supporting its prebiotic activity and its potential role in modulating the gut microbial community.

Overall, the results indicate that dietary supplementation with NT and MOS, particularly when used in combination, may enhance the economic viability of *P. vannamei* production in synbiotic culture systems. Nevertheless, further studies are required to clarify the biological significance of the observed gut morphological changes and to better understand the interactions between these additives and the gut microbiota.

## Figures and Tables

**Table 1:** Nutritional composition of the control and experimental diets used for juvenile *P. vannamei* cultured in a synbiotic system for 60 days.

	Diet Composition			
Ingredients (g kg^−1^)	Control	NT	MOS	NT/MOS
Wheat flour ^a^	120	118	118	118
Soybean meal ^b^	220	220	220	220
Meat and bone meal ^c^	120	120	120	120
Poultry by-product ^d^	120	120	120	120
Broken rice ^e^	138.1	138.1	138.1	138.1
Rice bran meal ^e^	40	40	40	40
Fish meal ^f^	60	60	60	60
Hemoglobin ^g^	62.6	62.6	62.6	62.6
Soy lecithin ^h^	5	5	5	5
Fish oil ^f^	15	15	15	15
Chicken oil ^i^	15	15	15	15
Potassium chloride ^j^	7	7	7	7
Salt	3.1	3.1	3.1	3.1
Magnesium oxide ^k^	12.34	12.34	12.34	12.34
Dolomite limestone ^l^	40	40	40	40
Vitamin and Mineral Supplement ^m^	5	5	5	5
DL-Methionine ^n^	1.86	1.86	1.86	1.86
L-Threonine ^o^	3.23	3.23	3.23	3.23
Nutribinder ^p^	6	6	6	6
Fylax (Antifungal) ^q^	3	3	3	3
Vitamin C (35%) ^r^	0.57	0.57	0.57	0.57
Emulsifying agent ^s^	2	2	2	2
Antioxidant Banox ^t^	0.2	0.2	0.2	0.2
NT ^u^	0	2	0	1
MOS ^v^	0	0	2	1
Proximal composition (Dry matter %)				
Moisture	8.13 ± 0.01	12.67 ± 0.007	13.75 ± 0.005	9.01 ± 0.01
Crude protein	37.73 ± 2.14	36.39 ± 1.74	37.87 ± 1.00	37.08 ± 0.94
Crude fat	5.99 ± 0.58	7.08 ± 0.08	6.67 ± 0.042	6.08 ± 0.04
Crude fiber	4.52 ± 0.59	4.29 ± 0.87	5.57 ± 0.95	6.12 ± 0.31
Ash	13.50 ± 0.01	13.30 ± 0.01	12.72 ± 0.002	13.20 ± 0.01

^a^ Cidade Bella Moinho, Ponta Grossa, PR, Brazil; ^b^ Cooperativa Comigo, Rio Verde, GO, Brazil; ^c^ Minerva S/A—Mirassol D’oeste, MT, Brazil; ^d^ Frango Rico, Votuporanga, SP, Brazil; ^e^ Dallas, Nova Alvorada do Sul, MS, Brazil; ^f^ BFP bioprodutos de pescado LTDA, Itajaí, SC, Brazil; ^g^ Hemoprot, Lins, SP, Brazil; ^h^ Adicel Indústria e Comércio—Ingredientes para Indústrias de Alimentos, Belo Horizonte, MG, Brazil; ^i^ Frango Rico, Votuporanga, SP, Brazil; ^j^ Brasil Química Ind. e Com. LTDA, Batatais, SP, Brazil; ^k^ Magnesium do Brasil AS, Fortaleza, CE, Brazil; ^l^ Mineracao João Vaz Sobrinho LTDA, Arcos, MG, Brazil; ^m^ De Heus nutrição animal, Rio Claro, SP, Brazil; ^n^ Rhodimet^®^ NP99 Adisseo a bluestar company, Beijing, China; ^o^ L-Threonine 98% Ajinomoto do Brasil Indústria e Comércio de Alimentos Ltd. a, Limeira, SP, Brazil; ^p^ Nutri-Bind Aqua Adisseo a bluestar company, Shanghai, China; ^q^ Selko Feed Aditives, Tilburg, The Netherlands; ^r^ Heilongjiang NHU Biotechnology CO. Ltd., Suihua, China; ^s^ Duas Rodas, Jaraguá do Sul, SC, Brazil; ^t^ Alltech, São Pedro do Ivaí, PR, Brazil; ^u^ Biotide Extra/Biorigin, Lençóis Paulista, SP, Brazil; ^v^ Hypergen/Biorigin, Lençóis Paulista, SP, Brazil.

**Table 2:** Water quality parameters of *P. vannamei* cultured fed diets supplemented with NT and MOS in a synbiotic system.

Parameters	RC	MOS	NT	NT/MOS
Dissolved oxygen (mg L^−1^)	6.60 ± 0.36	6.56 ± 0.35	6.56 ± 0.38	6.58 ± 0.37
Temperature (°C)	27.19 ± 0.76	27.19 ± 0.75	27.19 ± 0.77	27.20 ± 0.78
Salinity (g L^−1^)	24.40 ± 0.78	24.55 ± 0.83	24.56 ± 0.78	24.56 ± 1.03
pH	7.64 ± 0.36	7.68 ± 0.34	7.65 ± 0.33	7.67 ± 0.34
TAN (mg L^−1^)	0.39 ± 0.34	0.42 ± 0.24	0.15 ± 0.25	0.31 ± 0.21
NO_2_-N (mg L^−1^)	0.51 ± 0.22	0.46 ± 0.30	0.52 ± 0.25	0.51 ± 0.29
NO_3_-N (mg L^−1^)	326.05 ± 274.81	308.55 ± 249.58	308.80 ± 249.16	269.49 ± 168.91
Orthophosphate (mg L^−1^)	16.67 ± 2.42	16.07 ± 4.49	16.85 ± 5.84	18.54 ± 2.89
Total Alkalinity (mg CaCO_3_ L^−1^)	150.53 ± 23.23	154.96 ± 24.49	153.03 ± 18.92	159.52 ± 25.72
SS (mg L^−1^)	5.66 ± 1.90	5.92 ± 2.55	5.40 ± 2.20	6.43 ± 1.84

The data represent means and standard deviations. The results were analyzed using repeated-measures ANOVA (parametric data) and the Kruskal–Wallis test (nonparametric data) (*p* < 0.05). A control diet without additives (RC); a diet supplemented with 2 g NT kg^−1^ (NT); a diet supplemented with 2 g MOS kg^−1^ (MOS); and a diet supplemented with 1 g NT kg^−1^ and 1 g MOS kg^−1^ (NT/MOS).

**Table 3:** Return on investment (ROI) and relative percentage difference in the use of MOS and nucleotides in *P. vannamei* feed in a synbiotic system.

			RC	NT	MOS	NT/MOS
Revenues and costs	Stocking	shrimp ha^−1^	800,000	800,000	800,000	800,000
	Survival	%	78.28	75.40	78.75	74.79
	Harvested shrimp	shrimp ha^−1^	626,240	603,360	630,000	598,320
	Shrimp final weight	g	11.48	12.53	11.77	12.33
	Harvested biomass	kg ha^−1^	7189	7560	7415	7377
	Revenues	USD ha^−1^	26,599	27,972	27,436	27,296
	FCR		1.66	1.69	1.68	1.53
	Feed supplied	kg ha^−1^	11,934	12,777	12,457	11,287
	Feed cost	USD ha^−1^	10,979	11,755	11,460	10,384
	Additive cost	USD ha^−1^	0	194	224	187
	Expenses	USD ha^−1^	10,979	11,949	11,685	10,572
Benefits	Net income	USD ha^−1^	15,621	16,024	15,751	16,724
	Comparison	USD ha^−1^		403	130	1104
	ROI	%		207.49	58.02	588.97
	Relative difference	%		2.58	0.83	7.06

A control diet without additives (RC); a diet supplemented with 2 g NT kg^−1^ (NT); a diet supplemented with 2 g MOS kg^−1^ (MOS); and a diet supplemented with 1 g NT kg^−1^ and 1 g MOS kg^−1^ (NT/MOS).

**Table 4:** Presumptive total count of *Vibrio* spp., *Bacillus* spp., and yeasts in the gut of *P. vannamei* fed experimental diets containing nucleotides and MOS during 60 days of culture in a synbiotic system.

		RC	MOS	NT	NT/MOS	*p* Value
0 Day	*Bacillus* spp.	2.86 ± 7.65	2.86 ± 7.65	2.86 ± 7.65	2.86 ± 7.65	-
	*Yeast* spp.	0.09 ± 0.09	0.09 ± 0.09	0.09 ± 0.09	0.09 ± 0.09	-
	*Vibrio* spp.	5.14 ± 2.99	5.14 ± 2.99	5.14 ± 2.99	5.14 ± 2.99	-
	Sucrose-positive (%)	100	100	100	100	-
	No Sucrose-positive (%)	0	0	0	0	-
30 Day	*Bacillus* spp.	28.40 ± 59.30 ^b^	92.70 ± 6.49 ^b^	18.40 ± 18.10 ^b^	132.00 ± 118.00 ^a^	0.0005
	*Yeast* spp.	0.128 ± 0.12 ^a^	0.169 ± 0.18 ^a^	0.0373 ± 0.33 ^b^	0.120 ± 0.10 ^a^	0.0281
	*Vibrio* spp.	45.4 ± 101.00 ^a^	1.39 ± 0.54 ^b^	0.962 ± 1.15 ^c^	45.5 ± 70.00 ^a^	0.0001
	Sucrose-positive (%)	78.39	77.56	46.39	56.21	-
	No Sucrose-positive (%)	21.61	22.44	53.61	43.79	-
60 Day	*Bacillus* spp.	4.59 ± 1.49 ^b^	13.9 ± 10.10 ^a^	4.42 ± 3.77 ^b^	7.15 ± 11.70 ^b^	0.0184
	*Yeast* spp.	0.137 ± 0.07	0.164 ± 0.08	0.117 ± 0.07	0.130 ± 0.08	0.7411
	*Vibrio* spp.	2.16 ± 4.01	0.12 ± 0.12	0.05 ± 0.05	0.169 ± 0.22	0.4713
	Sucrose-positive (%)	75.00	83.60	83.33	63.54	-
	No Sucrose-positive (%)	25.00	16.40	16.67	36.46	-

The data are presented as means and standard deviations (CFU g^−1^ × 10^7^). The results were analyzed using the Kruskal–Wallis test (*p* < 0.05), followed by the Student–Newman–Keuls post hoc test. Mean values within the same row followed by different superscript letters indicate significant differences among treatments. A control diet without additives (RC); a diet supplemented with 2 g NT kg^−1^ (NT); a diet supplemented with 2 g MOS kg^−1^ (MOS); and a diet supplemented with 1 g NT kg^−1^ and 1 g MOS kg^−1^ (NT/MOS).

**Table 5:** Histomorphology of the anterior and mid gut of *P. vannamei* fed experimental diets supplemented with NT and MOS for 60 days.

Morphology		Treatments				
		RC	MOS	NT	NT/MOS	*p* Value
Foregut	Fold height	22.5 ± 7.6 ^a^	16.1 ± 3.1 ^c^	20.5 ± 4.7 ^ab^	16.0 ± 4.6 ^c^	<0.0001
	Fold width	24.6 ± 8.3 ^a^	20.03 ± 5.8 ^abc^	22.8 ± 6.6 ^ab^	17.2 ± 3.8 ^c^	<0.0017
	Gut wall thickness	35.5 ± 7.3 ^c^	53.0 ± 18.9 ^ab^	40.6 ± 9.4 ^bc^	62.4 ± 20.8 ^a^	<0.0001
Midgut	Fold height	15.9 ± 3.3 ^b^	19.6 ± 3.9 ^a^	16.2 ± 3.3 ^b^	13.9 ± 3.5 ^b^	<0.0001
	Fold width	26.9 ± 6.9 ^a^	22.3 ± 5.9 ^ab^	18.9 ± 6.2 ^b^	22.4 ± 6.1 ^ab^	<0.0003
	Gut wall thickness	28.5 ± 9.4 ^c^	52.7 ± 15.9 ^a^	42.2± 14.6 ^ab^	38.3 ± 9.9 ^b^	<0.0001

Data are expressed as the mean (*n* = 3 shrimp gut per tank) ± standard deviation. Results were analyzed using the nonparametric Kruskal–Wallis test (*p* < 0.05), followed by Dunn’s post hoc test with Bonferroni correction. Means in the same row with different superscripts differ significantly. A control diet without additives (RC); a diet supplemented with 2 g NT kg^−1^ (NT); a diet supplemented with 2 g MOS kg^−1^ (MOS); and a diet supplemented with 1 g NT kg^−1^ and 1 g MOS kg^−1^ (NT/MOS).

## Data Availability

The original contributions presented in this study are included in the article. Further inquiries can be directed to the corresponding author.
